# Virtual Reality as Tool for Bioprinting Quality Inspection: A Proof of Principle

**DOI:** 10.3389/fbioe.2022.895842

**Published:** 2022-06-09

**Authors:** Sarah Gretzinger, Barbara Schmieg, Gisela Guthausen, Jürgen Hubbuch

**Affiliations:** ^1^ Institute of Functional Interfaces, Karlsruhe Institute of Technology (KIT), Karlsruhe, Germany; ^2^ Institute of Engineering in Life Sciences, Section IV: Molecular Separation Engineering, Karlsruhe Institute of Technology (KIT), Karlsruhe, Germany; ^3^ Institute of Mechanical Process Engineering and Mechanics, Karlsruhe Institute of Technology (KIT), Karlsruhe, Germany; ^4^ Engler Bunte Institute Water Chemistry and Technology, Karlsruhe Institute of Technology (KIT), Karlsruhe, Germany

**Keywords:** virtual reality (VR), bioprinting, quality inspection, magnetic resonance imaging (MRI), cell distribution

## Abstract

As virtual reality (VR) has drastically evolved over the past few years, the field of applications of VR flourished way beyond the gaming industry. While commercial VR solutions might be available, there is a need to develop a workflow for specific applications. Bioprinting represents such an example. Here, complex 3D data is generated and needs to be visualized in the context of quality control. We demonstrate that the transfer to a commercially available VR software is possible by introducing an optimized workflow. In the present work, we developed a workflow for the visualization of the critical quality attribute (cQA) cell distribution in bioprinted (extrusion-based) samples in VR. The cQA cell distribution is directly influenced by the pre-processing step mixing of cell material in the bioink. Magnetic Resonance Imaging (MRI) was used as an analytical tool to generate spatially resolved 2.5 and 3D data of the bioprinted objects. A sample with poor quality in respect of the cQA cell distribution was identified as its inhomogeneous cell distribution could be displayed spatially resolved in VR. The described workflow facilitates the usage of VR as a tool for quality inspection in the field of bioprinting and represents a powerful tool for visualization of complex 3D MRI data.

## Introduction

Virtual Reality (VR) is an emerging technology for the visualization and communication of scientific data as well as process planning. With digital twins, “spatial relationships” (Mojica et al., 2021) or 3D movements can be experienced in a fast way from different perspectives. Examples can be found in medicine, where tomography images and their 3D reconstructions of the individual patient are used in combination with physical models for preceding surgery simulation and training (Mojica et al., 2021; Raimondi et al., 2021). Similarly, dynamic processes or the interaction of forces that were calculated in computed fluid dynamics (CFD) simulations can be accessed (Solmaz and van Gerven, 2021). For the communication with the public, architects, historians and archaeologists visualize construction plans, research on sceneries from the past as well as reconstructions of destroyed cultural heritage objects (Fried et al., 2020; Midtbø and Harrie, 2021).

In the field of life sciences imaging and visualization of complex structures is commonly performed. Thus, VR represents an interesting tool for life science applications in terms of visualization and communication (Stefani et al., 2018). An advantage of VR is, by adding transparency to a model of a complex structure, that surface properties and inner geometries or specifically marked locations can be inspected simultaneously. In contrast to traditional workflows based on a large number of 2D sections, VR allows scientists to get into touch with the holograms in a virtual room and mark 3D regions of interest intuitively (Schindler et al., 2017; Midtbø and Harrie, 2021). The digital twins can be shared and virtually discussed by teams, whose members physically are at different locations. Furthermore the possibility of interactive communication, trainings, and presentations is provided (Cassidy et al., 2020). The scientific discussion facilitates error detection and the planning of complex processes, as well as process control and optimization (Luecking et al., 2015; Cassidy et al., 2020).

In the life science discipline bioprinting complex 3D structures are manufactured making this scientific field a strong candidate for using VR. In bioprinting, the reproducible manufacturing of tissue models for cellular high throughput screenings may improve quality and efficacy of drug and medicinal product development processes (Gretzinger et al., 2018; Bowser and Moore, 2020). Within these model geometries, the localization of cells embedded is fundamental for process understanding and quality control. Based on a digitally designed geometry, bioprinters dispense filaments or a series of droplets of the so-called bioink in a layer-by-layer process generating a complex 3D object with spatially defined positioning. Hereby the bioink contains cells and other components forming a hydrogel around them to mimic natural tissue. Preparing the bioink by mixing is predominantly a manual process with small batches, as the components are expensive and the amount of cells is often limited (Gillispie et al., 2020; Pössl et al., 2021), discouraging material loss in devices or during transfer operations. In literature, the use of single-use static mixers or reciprocating movements between two vessels with a bottleneck in between are state of the art (Puertas-Bartolomé et al., 2020; Silva et al., 2020). Some complex solutions are under investigation, for example, active mixers (Ortega et al., 2019; Wenger et al., 2020; Dani et al., 2021).

The aims of the preparation steps including mixing and the filling of a single-use cartridge for bioprinting are to disperse the cells homogeneously in the bioink and avoid clogging of the bioprinting system (Ferris et al., 2013) while maintaining their viability. However, gradients can be caused by incomplete mixing, for example, due to dead volumes in the devices, density gradients or incomplete mixing caused by too short mixing times.

Inhomogeneous distribution of the cells within the bioink by a suboptimal mixing process will create regions with low cell density in the bioprinted object. As tissue formation is dependent on cell-to-cell communication and thus requires a sufficiently high concentration, analyzing the localization of the cells within the bioprinted objects is decisive for their quality and thus, the future application within patients.

As a destructive measurement or sampling is not possible, tracking of the cells within the printed geometries and therewith, quality control at certain timepoints of the product life cycle is crucial for the transit to industrial scale, Process Analytical Technology (PAT) and standardization (An et al., 2021; Strauß et al., 2021; Uzun-Per et al., 2021). The methods used for this analysis must be destruction-free and gentle. Magnetic Resonance Imaging (MRI), which is commonly used in medicine, meets these requirements. In contrast to confocal microscopy, which has a limited penetration depth for non-transparent materials, MRI measures the nuclear spins, their distribution and properties in 1D–3D (Callaghan, 1991; Kimmich, 1997). Labelling cells with biocompatible contrast agents allows the segmentation from surrounding hydrogel without bleaching effects that limit long-term monitoring in light microscopy of cells and cell aggregates (Billotey et al., 2003; Estelrich et al., 2015; Busato et al., 2016; Pössl et al., 2021). One example for well suited MRI-contrast agents are nano-sized magnetic iron oxide particles, which mainly reduce *T*_2_ relaxation of labelled cells in comparison to unlabeled cells or hydrogel (Souza et al., 2010).

In the field of bioprinting the location of cells within bioprinted objects has a direct impact on the quality. During the manufacturing steps of a bioprinting process, the reproducibility and the performance, as already mentioned, of the mixing step has direct impact on the location of cells within the bioprinted object and thus, its quality. The visualization in a VR environment gives a fast overview and highlights local maxima and minima that may be critical. In comparison to the analysis of 2D cross-sections and threshold diagrams, the method may be suited for on-time process control and used for fast decision-making in process optimization.

**FIGURE 1:**
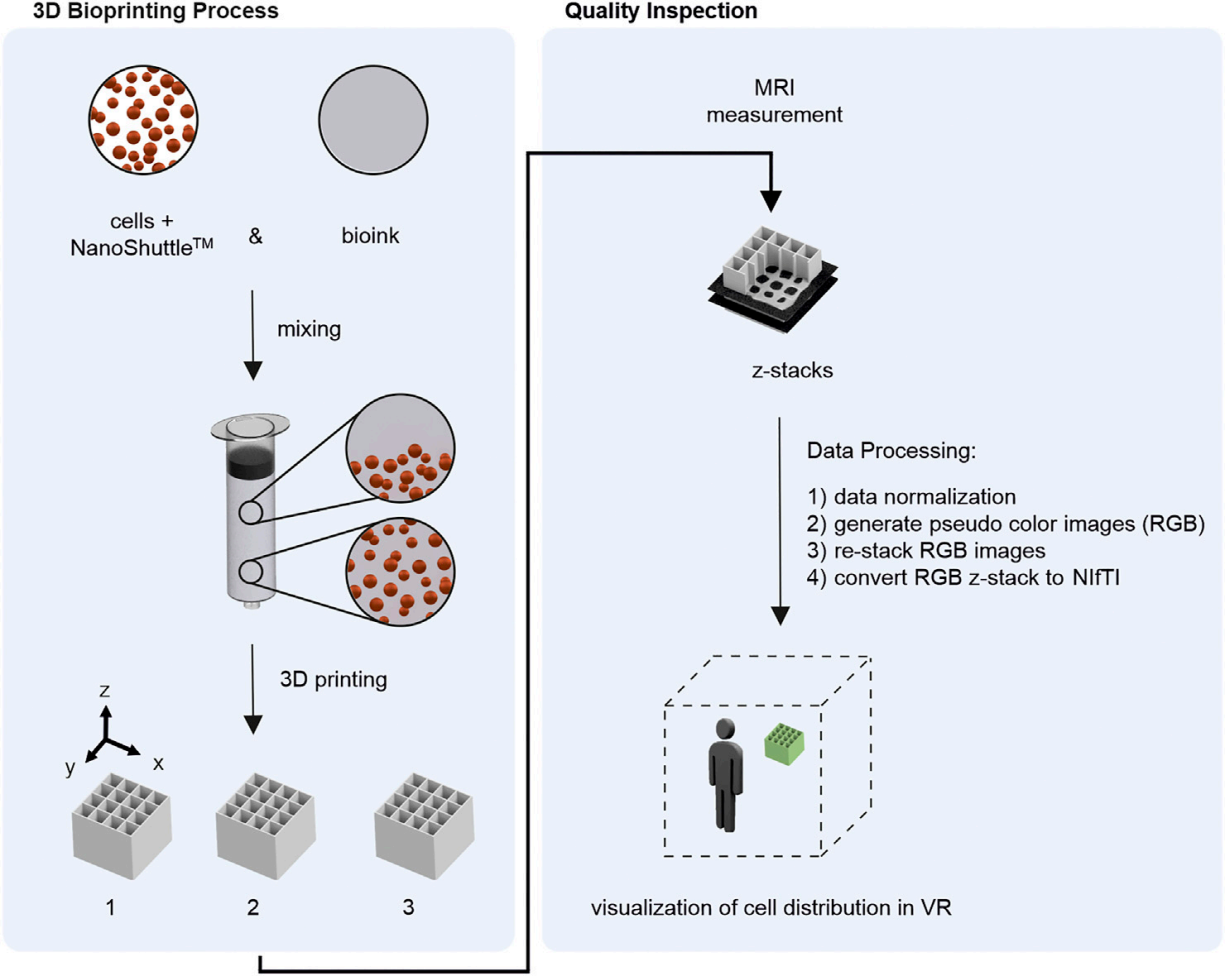
Overview of the developed workflow. Left) 3D Bioprinting process including mixing magnetically tagged cells (using NanoShuttle™) with bioink and consecutive 3D printing of the material to cubes. Bioprinting was done with 3.3 ml bioink-cell-mixture resulting in 3 objects per run. The experiment was performed 4 times (*n* = 4) resulting in 3 × 4 = 12 samples for quality inspection. Right) Quality Inspection starting with MRI measurement of each sample resulting in z-stacks with 15 axial slices. After four steps of data processing the MRI data could be inspected in a VR environment.

In the present study, a workflow for visualization of the cell distribution within bioprinted samples in VR was developed and evaluated. The development includes the extension of the previously developed MRI method (Schmieg et al., 2022) toward the detection of cells using magnetic tags and the transfer of the generated MRI data to a VR environment. In a first case study, quality inspection of the critical quality attribute (cQA) cell distribution using VR was done. The cQA cell distribution was chosen since the pre-processing step mixing cell material in the bioink has a direct influence on the cQA. Firstly, magnetically-tagged cells were mixed with bioink using a static mixer prior to the bioprinting process. Secondly, the bioprinted samples were measured by MRI and the MRI data were transferred to VR. For VR visualization the software ConfocalVR 3.2 (Immersive Science LLC, Newcastle, WA, United States) (Stefani et al., 2018) was used. Therefore, a four-step data processing step needed to be introduced since the software was developed for confocal microscopy images. Overall, we show that the here described workflow enables VR visualization of complex bioprinting data showing the power and usefulness of VR as a tool for quality inspection in the field of bioprinting. The experimental and analytical workflow is summarized in Figure 1.

## Materials and Methods

### Cell Culture

The fibroblast cell line NIH-3T3 was purchased form CLS (Cell Line Services GmbH, Eppenheim, Germany). Cell culture reagents were purchased from Thermo Fisher Scientific if not stated otherwise. Cultivation was done in Dulbecco’s Modified Eagle Medium (DMEM) supplemented with 10% fetal bovine serum (FBS) and 1% Penicillin/Streptomycin at 37°C in a humidified 5% CO_2_ incubator. The cell culture was split every 7 days to a concentration of 1 × 10^4^ cells/cm^2^ with 1 medium change after 3 days.

Magnetic cell labelling for MRI imaging was done using T25 flasks; 0.8 ml of NanoShuttle-PL (Greiner Bio One, Monroe, NC, United States) contrast agent and 0.25 × 10^6^ cells NIH-3T3 fibroblasts were suspended in 5 ml medium and incubated for 5 days (37°C, 5% CO_2_, 95% relative humidity), which led to an uptake of NanoShuttle-PL into the cells. For bioprinting, the trypsinized (PBS) cell suspension was purified using a MagRack (Cytiva), which retained tagged cells. The purified material was resuspended in 0.6 ml of fresh cell culture medium.

### Bioink Preparation and Bioprinting

One cartridge of bioink for bioprinting consisted of 0.3 ml of the aforementioned cell suspension and a commercially available alginate—nanocellulose blend (Cellink Bioink, Cellink AB, Gothenburg, Sweden). The components were mixed with a single-use static mixer (Cellmixer, Cellink AB) which is recommended (specifically optimized) for mixing 3 ml of the high-viscous alginate—nanocellulose material with 0.3 ml of cell suspension. Two cartridges containing the respective components are fixed within the device to enable plunger movement synchronization. The mixing process is controlled by manual pressure of the operator. The freshly mixed bioink was dispensed directly into the barrel tip opening of a 3 ml single-use cartridge (Nordson Corporation, Westlake, United States) for bioprinting.

Bioprinting was executed with an extrusion-based system (3D Discovery, regenHU, Villaz-St-Pierre, Switzerland) equipped with a pneumatic printhead and a conic nozzle (inner diameter 0.25 mm) as described in (Schmieg et al., 2022). In short, meandering strands were extruded to form a cubic scaffold with dimensions of 8.8 mm × 8.8 mm × 8 mm (5 strands with a distance of 2.2 mm, layer height 0.20 mm) with a pressure of 20 kPa and a velocity of 20 mm/s at 23°C. Directly after the printing, the objects were crosslinked with CaCl_2_-solution (Cellink AB) for 20 min (Schmieg et al., 2022) and stored at room temperature in a 10 ml vial for a maximum of 2.5 h with closed lid before MRI measurement. Three objects could be 3D printed with 3.3 ml of bioink starting material (n_scaffolds_per_cartridge_ = 3). The experiment was conducted with four cartridges (n_cartridge_ = 4), which yielded a total number of n_scaffolds, cell_ = 4 × 3 = 12 cubic scaffolds. As reference objects, two scaffolds were printed without cell suspension (n_cartridge_ = 1, n_scaffolds_per_cartridge_ = 2, n_scaffolds, reference_ = 2). Hereby, one cartridge of alginate—nanocellulose blend was used as received. All experiments were conducted using the same batch of the ink.

### Magnetic Resonance Imaging Measurement

The cubic scaffolds were imaged with an Avance HD III SWB 200 MHz tomograph (Bruker BioSpin GmbH, Ettlingen, Germany) equipped with a 20 mm birdcage (MICWB40, Bruker) with air as background medium at 20°C as described in (Schmieg et al., 2022). With pulse sequence rapid acquisition with relaxation enhancement (RARE) (Callaghan, 1991; Kimmich, 1997; Bernstein et al., 2004), a stack of 15 axial slices was measured, which resulted in a measurement time of 26 min. The measured data matrix of NMR-intensities is strongly weighted with the transverse relaxation time *T*_2_ which strongly depends on the material composition within the individual voxel. Thereby, the matrix of each of the 15 axial slices consisted of 256 × 256 pixels with a slice thickness of 0.4 mm and a total interslice distance of 0.5 mm. The field of view was chosen either 12 mm^2^ × 12 mm^2^, 14 mm^2^ × 14 mm^2^ or 16 mm^2^ × 16 mm^2^, depending on the swelling and positioning of the 3D printed scaffold within the tomograph.

### 2D Image Analysis

Image analysis of the slices was done in MATLAB 2020a (The MathWorks, Inc., Natick, United States). Pixel intensity was normalized within the whole MRI data set (n_scaffolds, total_ = n_scaffolds, cell_ + _nscaffolds, reference_ = 14) with regard to respective field of view of the individual sample. A histogram of the intensity values of all 15 slices per object was calculated with the command “histogram” and a Bin Width of 1 for each bioprinted scaffold. The intensity scale can be projected to any false color scale and of course also to RGB (red green blue scale). In Supplementary Table S1 an example allocating the used commands is listed. To note: the individual form of the code options is depending on the MATLAB version and installed toolboxes.

### Data Processing and Virtual Reality Visualization

Image processing for VR consisted of four consecutive subprocesses: Data normalization, the conversion to RGB pseudo color images, 3D reconstruction by the re-stacking of RGB images and finally, the data formatting to generate a NIfTI file suitable for VR visualization using the Software ConfocalVR 3.2 (Immersive Science LLC, Newcastle, WA, United States) (Stefani et al., 2018) in combination with VR goggles. Using RGB color images is necessary when working with the commercial software ConfocalVR.

In the first data processing step, data were normalized as described in chapter 2.4. Background pixels were removed for better 3D visualization. Secondly, RGB pseudo color images were generated for each axial slice using the jet colorbar. The same limits for the jet colorbar need to be applied for each pseudo color image. These RGB images were re-stacked in z direction in the third step and consecutive converted to NIftI using ImageJ National Institutes of Health (NIH), Bethesda, MD; available on (https://imagej.nih.gov/ij/) as described in (Stefani et al., 2018). In Supplementary Table S2 an example allocating the used commands is listed. To note: the individual form of the code options is depending on the MATLAB version and installed toolboxes.

VR visualization of the MRI data was done using the Software ConfocalVR 3.2 (Immersive Science LLC, Newcastle, WA, United States) (Stefani et al., 2018) in combination with HTC Vive (HTC Corporation, Taoyuan City, Taiwan) VR goggles. The Host-PC was running Windows 10 Pro (processor Intel Core i7-6700 3.4/3.4 GHz; RAM 32 GB) with an NVIDIA GeForceGTX 1080.

The alginate-cellulose blend has a high water content and is characterized of high signal (pseudo color: red) intensity in the pseudo color image data. In the alginate-cellulose blend mixed with NanoShuttle-PL *T*_2_ relaxation is faster yielding a lower signal intensity (pseudo color: blue). The aim of a quality inspection was to visualize deviations that represent noncomplete mixing or phase separations within the material. The software ConfocalVR 3.2 (Immersive Science LLC, Newcastle, WA, United States) allows to display each channel (red green blue) separately or in any combination. This feature was used for the VR quality inspection of the MRI data.

### Software

The 3D Discovery was controlled by the HMI (Human Machine Interface) Software (regenHU 3D Discovery 8.23.9.38, regenHU, Villaz-St-Pierre, Switzerland). Scaffold design and G code generation was done using the BioCAD software (regenHU, Villaz-St-Pierre, Switzerland). MRI was operated with ParaVision 6.0.1 (Bruker BioSpin MRI GmbH). Data processing and plotting was performed with MATLAB R2020a (The MathWorks, Inc., Natick, United States). ImageJ software [National Institutes of Health (NIH), Bethesda, MD] was used for conversion of pseudo color z-stacks to NIftI file format as described in (Stefani et al., 2018). VR visualization of the MRI data was done using the Software ConfocalVR 3.2 (Immersive Science LLC, Newcastle, WA, United States) (Stefani et al., 2018). The video was recorded using NVIDIA^®^ GeForce^®^ Experience™ Version 3.23.0.74 and put together using DaVinci Resolve 17 (Blackmagic Design Pty. Ltd., Port Melbourne, Australia). Single frame isolation (taking snap shots) was also performed using DaVinci Resolve 17 (Blackmagic Design Pty. Ltd., Port Melbourne, Australia).

## Results

In the present work, a workflow was developed for the exploration of virtual reality (VR) as a tool for quality inspection in the field of 3D bioprinting, more specific, extrusion-based bioprinting. Here, mixing cell material with the viscous biomaterial represents a pre-processing step directly influencing the critical quality attribute (cQA) of cell distribution. The mixing process can result in: 1) homogeneous cell distribution 2) inhomogeneous cell distribution 3) without cell material (mixing step not performed); while only a homogeneous cell distribution is acceptable in terms of process performance. The presented concept visualizes spatial cell distribution of 3D bioprinted objects and allows interaction between several operators and the object in question in a VR environment for quality inspection of the cQA cell distribution. The experimental and analytical workflow is briefly shown in Figure 1. First, the magnetically tagged cells (using NanoShuttle-PL) were mixed with the bioink in a pre-processing step prior to the 3D printing of the blended material. The experiment was performed 4 times, in each run 3 objects were printed (n_samples_ = 4 × 3 = 12). For each sample a MRI scan was performed. In order to prepare the MRI data for VR, a four-step data processing procedure was performed.

### Image Analysis of Magnetic Resonance Imaging Slices

In general, bioinks used in the field of bioprinting need to be biocompatible. While each application has different requirements towards the bioink properties, the need for a high water content is universal for most cell applications. The commercially available bioink used in the present work is a representative of an alginate-cellulose blend bioink with a high water content (Markstedt et al., 2015) and is of high MRI signal intensity. As a reference for MRI signal intensity, two 3D printed objects only consisting of bioink without cells were analyzed. Additional control samples of homogenized biomaterial, ink mixed with cell culture medium as well as bioink starting material containing non-tagged cells can be found in the Supplementary Data S1. Experimentally, the water content of the control samples is not only dependent on the material composition, but also on the treatment with the crosslinking solution causing swelling. Additionally, drying takes place during handling. While this individual water content might influence the MRI signal, the overall signal reduction of the *T*_*2*_ contrast caused by the magnetic contrast agent is far more pronounced in the chosen MRI settings than the influence of the water signal.

**FIGURE 2:**
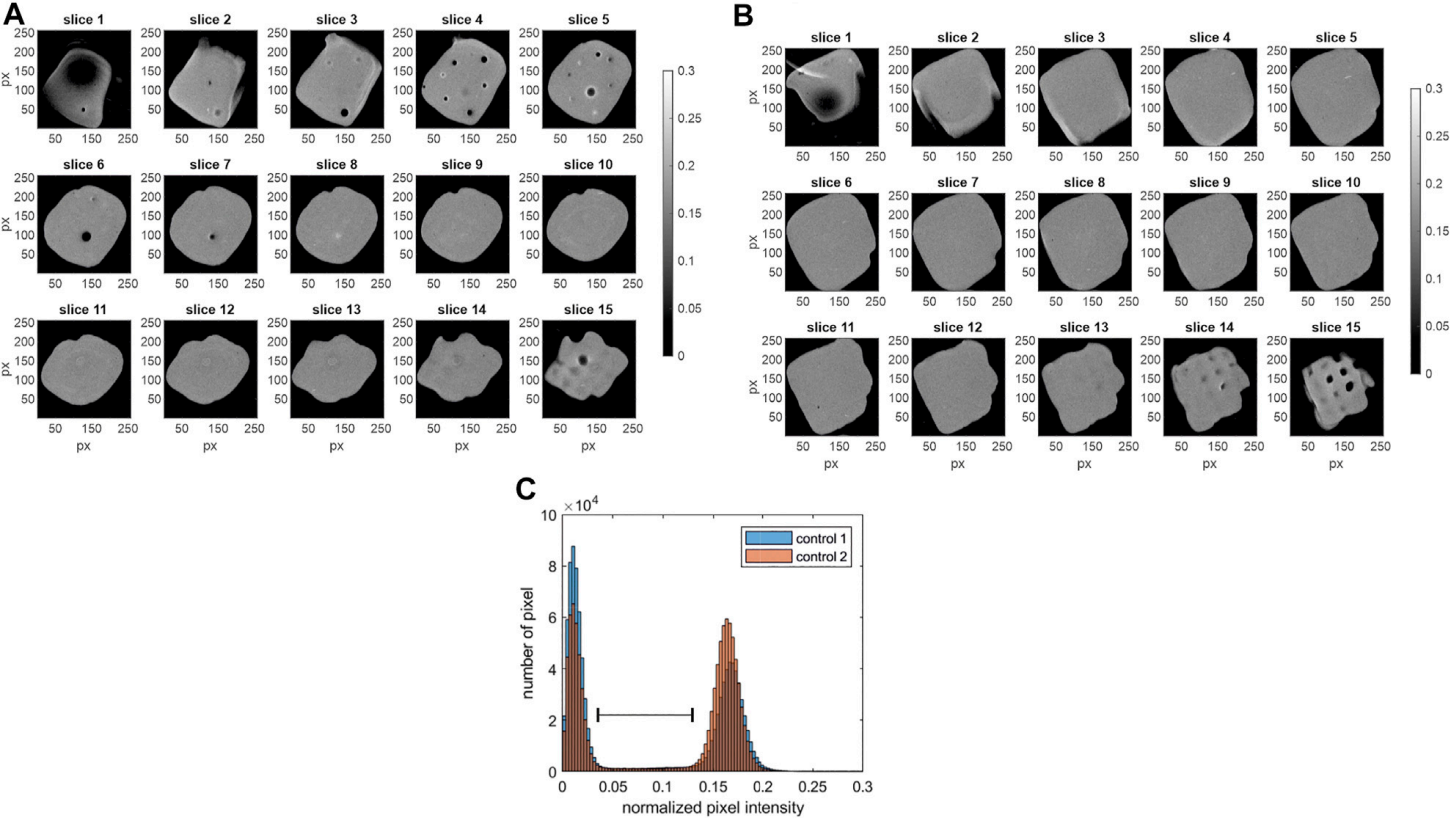
MRI data of the control samples. Shown are the grey scale images of the 15 axial slices (z-stack) with slice 1 as bottom and slice 15 as top slice (256 × 256 px). **(A)** control 1, field of view 12 mm^2^ × 12 mm^2^ **(B)** control 2, field of view 12 mm^2^ × 12 mm^2^. **(C)** Additionally, the histogram of the normalized pixel intensity (including all 15 slices of each MRI data set) is shown for both control samples. Low normalized pixel intensity <0.05 characterizes dark pixels in the images **(A,B)**. High pixel intensities representing the hydrogel areas (0.12 < I < 0.23) are displayed in bright colors. The black spacer visualizes the expected range of pixel intensity, where peaks of the samples containing cells with *T*_*2*_ contrast agents are expected.

Traditional analysis strategies include the visualization of 2D slices as well as histograms. The results are shown in Figure 2. Depicted are the grey scale images of the 15 axial z-slices (slice 1 = bottom slice; slice 15 = top slice). Control 1 is shown on the left (Figure 2A) and control 2 on the right (Figure 2B). Additionally, the normalized pixel intensity histogram of each MRI data set (including all 15 slices) is shown in Figure 2C. The images as well as the histogram display the possibility to segment the data into dark background pixels and bright pixels of the hydrogel. The normalized pixel intensity signal from process samples is expected in a lower range (indicated with black spacer), caused by the faster *T*_2_ relaxation of magnetically tagged cells. It is important to note that the MRI intensity profile might differ between different batches of bioink (Cellink Bioink, Cellink AB, Gothenburg, Sweden). Thus, controls and samples need to be from the same bioink (Cellink Bioink, Cellink AB, Gothenburg, Sweden) batch.

**FIGURE 3:**
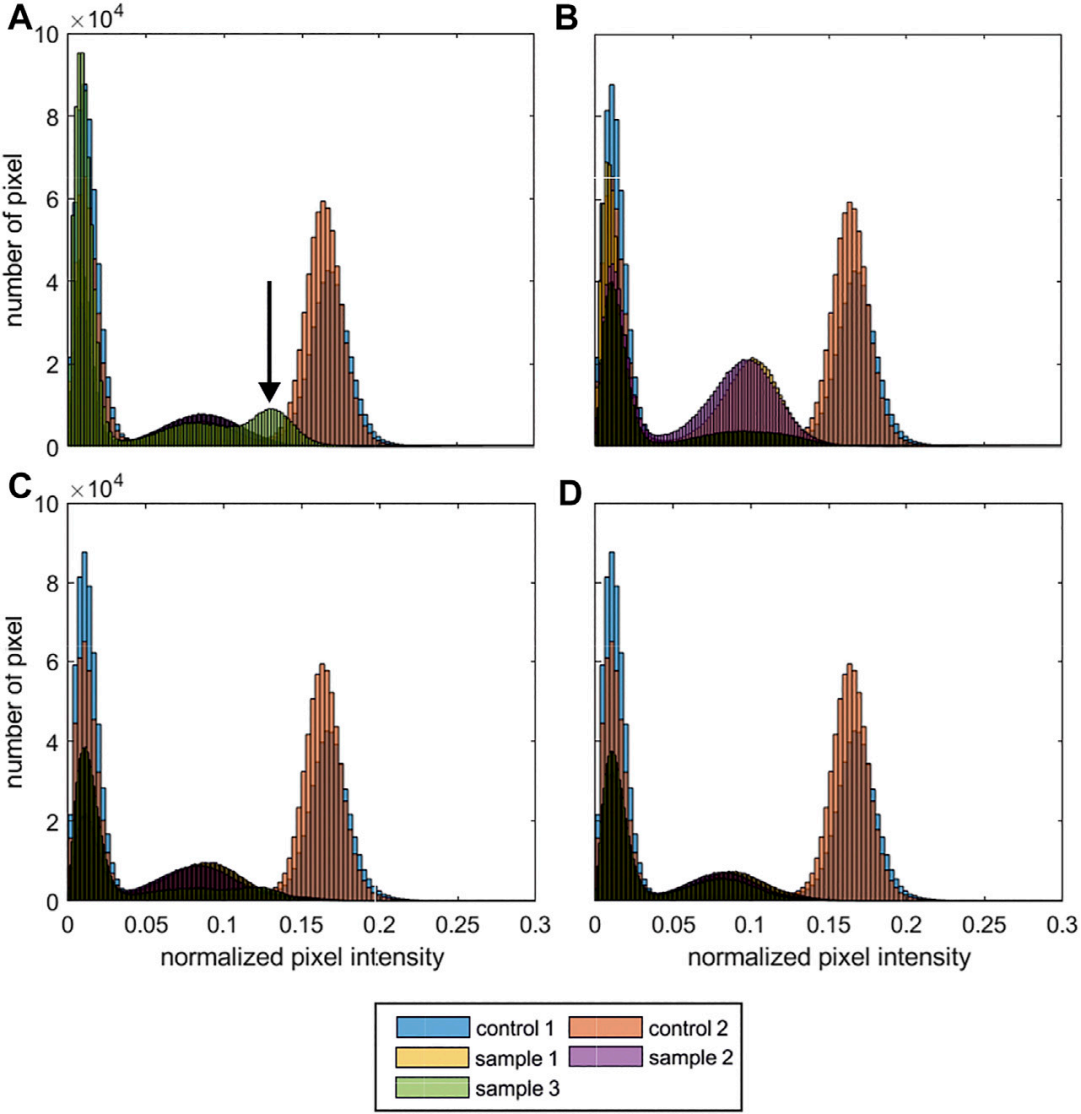
MRI histograms of the process samples. Shown are the histograms of the normalized pixel intensity (including all 15 slices of each MRI data set). For each run 3 objects were produced and analysed. For comparison the control samples 1 + 2 (Figure 2) are shown in each sub-figure. **(A)** run 1; **(B)** run 2; **(C)** run 4; **(D)** run 4. (n_scaffolds, cell_ = 4 × 3 = 12 cubic scaffolds; n_scaffolds, reference_ = 2 cubic scaffolds) The arrow indicates an anomalous normalized pixel intensity profile.

The histograms of the normalized MRI signal of the process samples are shown in Figure 3. For each process run 3 objects were produced and analysed (n_samples_ = 4 × 3 = 12). The MRI data of each run is shown separately (Figures 3A–D), for comparison the histograms of the control samples are shown as an overlay in each subfigure. While the histograms of 11 out of 12 samples is comparable (Figures 3B–D) indicating a reproducible processing, sample 3 of run 1 showed an anomalous intensity profile with an additional peak Figure 3A; indicated with arrow. Subsequently, only the MRI data of run 1 and control 1 are discussed in detail. To provide the complete dataset of the study, the grayscale images of the 15 axial z-slices of the inconspicuous process samples from run 2–4 are attached in the Supplementary Datas S2–S4, control samples were discussed in Figure 2.

**FIGURE 4:**
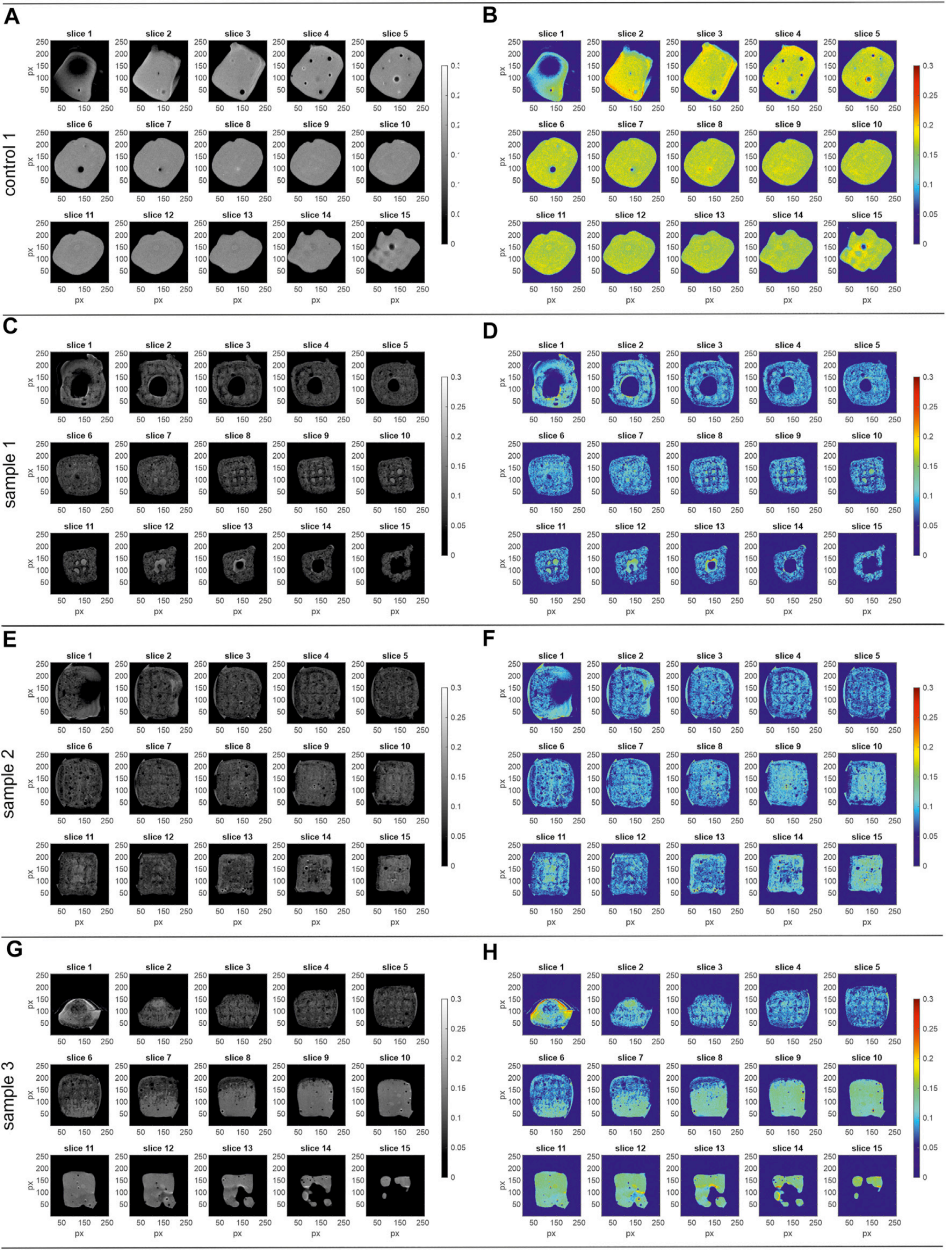
MRI data of the process samples run 1. Shown are the images of the 15 axial slices (z-stack) with slice 1 as bottom and slice 15 as top slice (256 × 256 px). Left) grey scale, right) pseudo colour images (RGB; jet colour bar) **(A,B)** control 1, field of view 12 mm^2^ × 12 mm^2^ **(C,D)** sample 1, field of view 16 mm^2^ × 16 mm^2^ **(E,F)** sample 2, field of view 16 mm^2^ × 16 mm^2^ **(G,H)** sample 3, field of view 16 mm^2^ × 16 mm^2^. The corresponding histograms of the normalized pixel intensity are shown in Figure 3A.

Figure 4 displays the 15 axial z-slice of the MRI data of run 1 that the histograms were calculated from. The figures show the grey scale images of the respective sample on the left while the corresponding pseudo color images (RGB; using the jet color bar) are on the right.

The control 1 sample (Figures 4A,B) shows higher MRI signal intensity than the process samples (run 1). While process sample 1 (Figures 4C,D) and sample 2 (Figures 4E,F) do not show any abnormalities throughout the slices with a reproducible signal intensity between the samples, sample 3 (Figures 4G,H) shows an inhomogeneous intensity profile over the 15 z-slices. While the signal intensity in slices 3–5 is similar to process samples 1 and 2, the signal intensity is gradually changing starting from slice 6 to the top of the object in slice 15. Whereas the intensity deviation occurs in a small area of slice 6, it is spread over the whole cross-section in the top slices, creating a three-dimensional volume in reality. Additionally, wrap-around artifacts at the bottom of the sample (Figures 4G,H; slice 1) can be observed.

### 3D Visualization

For 3D visualization in VR using ConfocalVR 3.2 (Immersive Science LLC, Newcastle, WA, United States) (Stefani et al., 2018), the pseudo color images were re-stacked and converted to NIftI file format. The virtual environment of ConfocalVR 3.2 offers the operator to rotate and inspect the imported representation of the 3D printed scaffold intuitively by observing the overall object with adaptable transparency and thresholds. Local anomalies can quickly be tagged and analyzed in detail. The software functions are described in detail in (Stefani et al., 2018).

**FIGURE 5:**
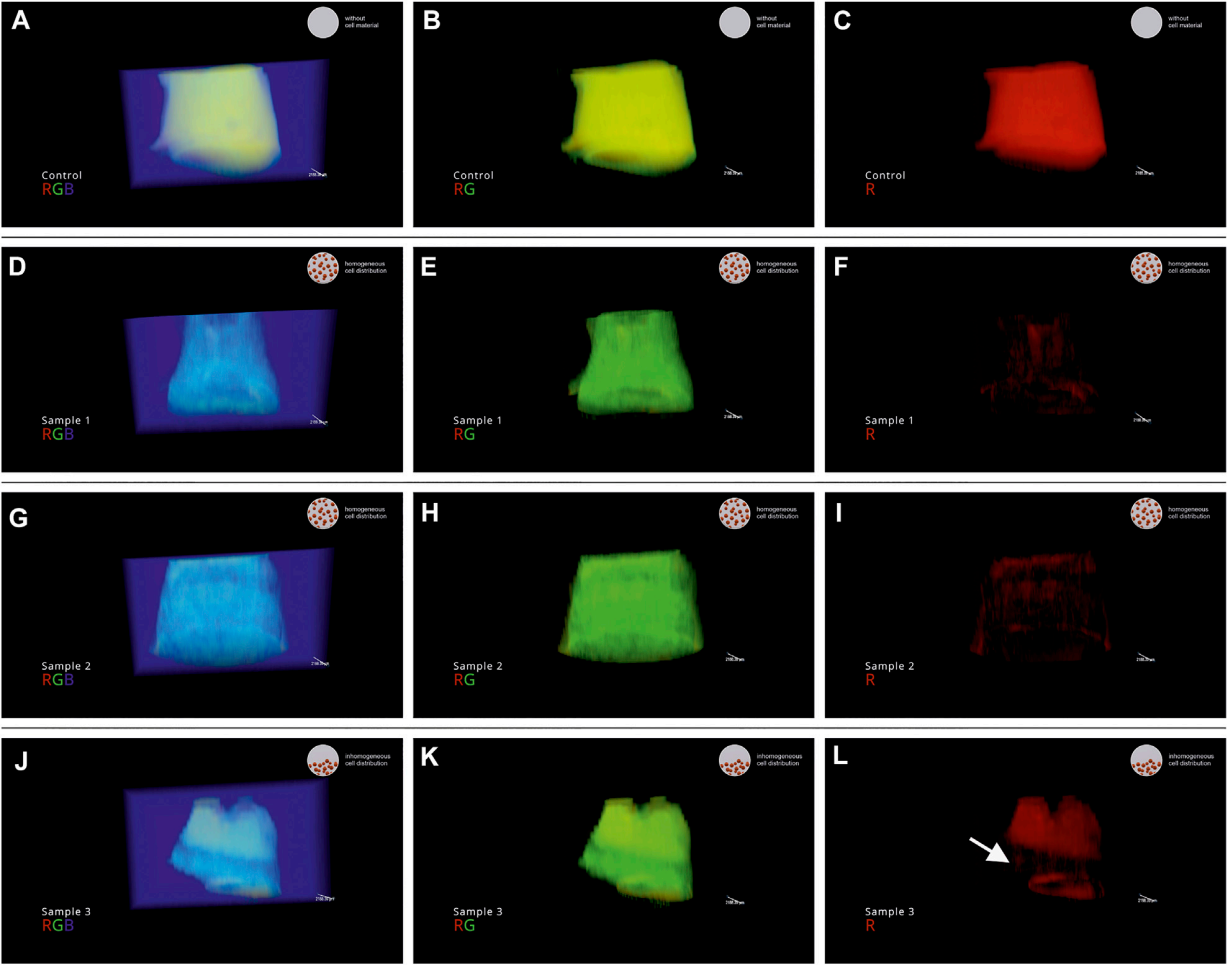
Snap shots of the recorded VR session of the process samples of run 1. **(A–C)** control 1; **(D–F)** sample 1 (run 1) **(G–I)** sample 2 (run 1) **(J–L)** sample 3 (run 1). In the video, the channels are visualized overlaid and subsequently switched off: displayed channels left) red, green, blue (RGB); middle) red, green (RG); right) red (R).

In the present work, VR was used as a tool for quality inspection of the cQA cell distribution in 3D. A video of a VR session was recorded and edited to explain the findings to the reader of this manuscript. Each sample (control 1, process samples run 1 No. 1–3, Supplementary Data S5) was visualized separately in rotation. To segment high and low signal intensities, the RGB channels are shown overlaid and subsequently switched off. Hereby, locations with certain signal intensities get visible and intuitive local thresholding is possible. The video was annotated for storytelling purpose. In the bottom left corner information on sample name and displayed channels are indicated. In the top right corner, the judgement of the operator on the cQA cell distribution is displayed: 1) homogeneous cell distribution 2) inhomogeneous cell distribution 3) reference without cell material (mixing step not performed). Single frames of the video were isolated as snap shots and displayed in Figure 5. Shown are three snap shots per sample, representing the overlaid and subsequently switched of channels (RGB): left) red, green, blue (RGB); middle) red, green (RG); right) red (R). In Figures 5A–C the snap shots of control 1 are shown where the commercial bioink was printed as received. Since the MRI signal intensity was high due to the high water content, the object is displayed in the red channel (Figure 5C). Subsequently, sample 1 (Figure 5F) and sample 2 (Figure 5I) are not displayed in the red channel due to decreased MRI signal intensity as seen in Figure 3A. Sample 3 showed an anomalous normalized pixel intensity profile (Figure 3A; indicated with arrow). Here, only the top half of sample 3 is displayed in the red channel (Figure 5L) indicating an inhomogeneous cell distribution with a low number of cells where a red signal can be detected.

## Discussion

MRI as a non-destructive analytical method in the field of bioprinting was discussed in a previous study (Schmieg et al., 2022), highlighting the importance to ensure quality and reproducibility when aiming towards industrial or clinical applications. While the previous work was focusing on analytical method development and the calculation of parameters describing the reproducibility of the bioprinting process, this study’s focus is on the development of a workflow for visualizing and communicating the complex 3D data. The presented strategy allows fast quality inspection and decision making in an VR environment that can be seen as an additional tool in comparison to the sequential analysis of 2D slices of the 3D object. As the focus of the case study was to transfer the data to VR in a setup suitable for quality inspection, commercially available entities for cell tagging and bioink pre-processing were used.

To localize cells within opaque 3D objects, the method used in the previous study (Schmieg et al., 2022) was adapted towards cell tracking. Using MRI in combination with magnetic particle-tags is commonly used for cell tracking purposes (Billotey et al., 2003; Tallheden et al., 2006; Bulte, 2009). In the present study, NanoShuttle™ was used. It is a commercial particulate formulation to magnetically tag cells for applications such as creating scaffold free spheres (Baillargeon et al., 2019; Bowser and Moore, 2020; Vu et al., 2021).

At the current state, the bioprinting process still includes several (semi)manual process steps. The pre-process step of mixing the bioink is an exemplary one of them. Here, magnetically tagged cells were mixed with bioink prior to the extrusion-based bioprinting process. Representing the execution of this pre-process step has direct influence on the cQA cell distribution of the finished printed object. Not focusing on the mixing process itself, a static mixer was used (Apelgren et al., 2017; Möller et al., 2017) as recommended by the manufacturer. Although using a commercial product the mixing process can result in the following cQAs: 1) homogeneous cell distribution 2) inhomogeneous cell distribution 3) without cell material (mixing step not performed). Hence, quality control should be performed as a homogeneous cell distribution needs to be achieved.

The bioprinting process described in Figure 1 was performed 4 times and analysed using MRI (n_samples_ = 4 × 3 = 12). As expected only a low number of samples (1 out of 12) showed an in-homogenous cell distribution due to the design of the case study. The sample with in-homogenous cell distribution was produced in run 1.

Subsequently, the printed objects from run 1 and a control were transferred in a VR environment for quality inspection. In this study, the VR software ConfocalVR 3.2 was used. The software is designed for confocal microscopy applications. However, by introducing the described data processing steps MRI data were successfully transferred in VR. In the present study, the function to display the different channels in overlay in any combination was extremely helpful for displaying the inhomogeneous cell distribution in object 3 of run 1. Magnetically tagged cells could only be found in the bottom half of the object. As a reminder, the bioink for each run was mixed with a static mixer filling a cartridge from the barrel tip opening. It is most likely that the mixing process was not performed properly at the beginning, resulting in non-mixed material at the top of the cartridge and thus inhomogeneous cell distribution in the last object printed of the cartridge. While the degree of freedom in VR is almost infinite, the presentation of the results in conventional media is severely limited. The strength of VR is the 3D visualization and direct interaction with a digital twin (Eiselt et al., 2013; Häfner et al., 2013; Haefner et al., 2014; Bellalouna, 2020). ConfocalVR 3.2 even allows for collaboration, meaning more than one person can be in the VR session (Stefani et al., 2018). This opens up the possibility for communication of complex 3D data which can be an advantage for interdisciplinary collaborations (Eiselt et al., 2013; Häfner et al., 2013) when quick and easy explanations are needed. In the field of Bioprinting (Malda et al., 2013), interactive discussions between the scientists specialized in cell culture, process engineering and application of the bioprinted objects, might enhance knowledge transfer and facilitate process optimization. In addition, different locations can easily work closely together.

## Conclusion and Outlook

In summary, a workflow for quality inspection of the cQA cell distribution of 3D bioprinted objects was developed. This was done based on a bioprinting case study where cells need to be mixed with bioink which has a direct influence of the cQA cell distribution. The workflow included magnetically tagging cells and, subsequently, performing an MRI scan of the printed objects. The MRI data could be transferred successfully in VR for quality inspection. It should be noted that while the focus of the present work was not on the experimental process itself, process optimization should be performed for every application regarding mixing performance and cell damage. In this manuscript manual inspection in a VR setting is done. Thus, it might not apply as tool for general process development at the moment when a high degree of automation is needed. However, it is an excellent way to present complex 3D data and represents an intuitive tool for quality inspection. The present case study highlighted the use of VR when an abnormal data set is spotted and 3D visualization could help to increase process understanding. VR even enables easy collaboration if shared VR sessions are possible. For these reasons, VR represents a powerful tool in quality inspection in the field of bioprinting and it holds the promise of investigating more complex structures with intrinsic structures or save digital twins as retention samples in future.

## Data Availability

The original contributions presented in the study are included in the article/Supplementary Material, further inquiries can be directed to the corresponding author.
