# Supplementary figures and images for: Virtual Reality as Tool for Bioprinting Quality Inspection: A Proof of Principle
Source: Front Bioeng Biotechnol. 2022 Jun 9;10:895842. doi: 10.3389/fbioe.2022.895842 (PMC9218671; doi:10.3389/fbioe.2022.895842)

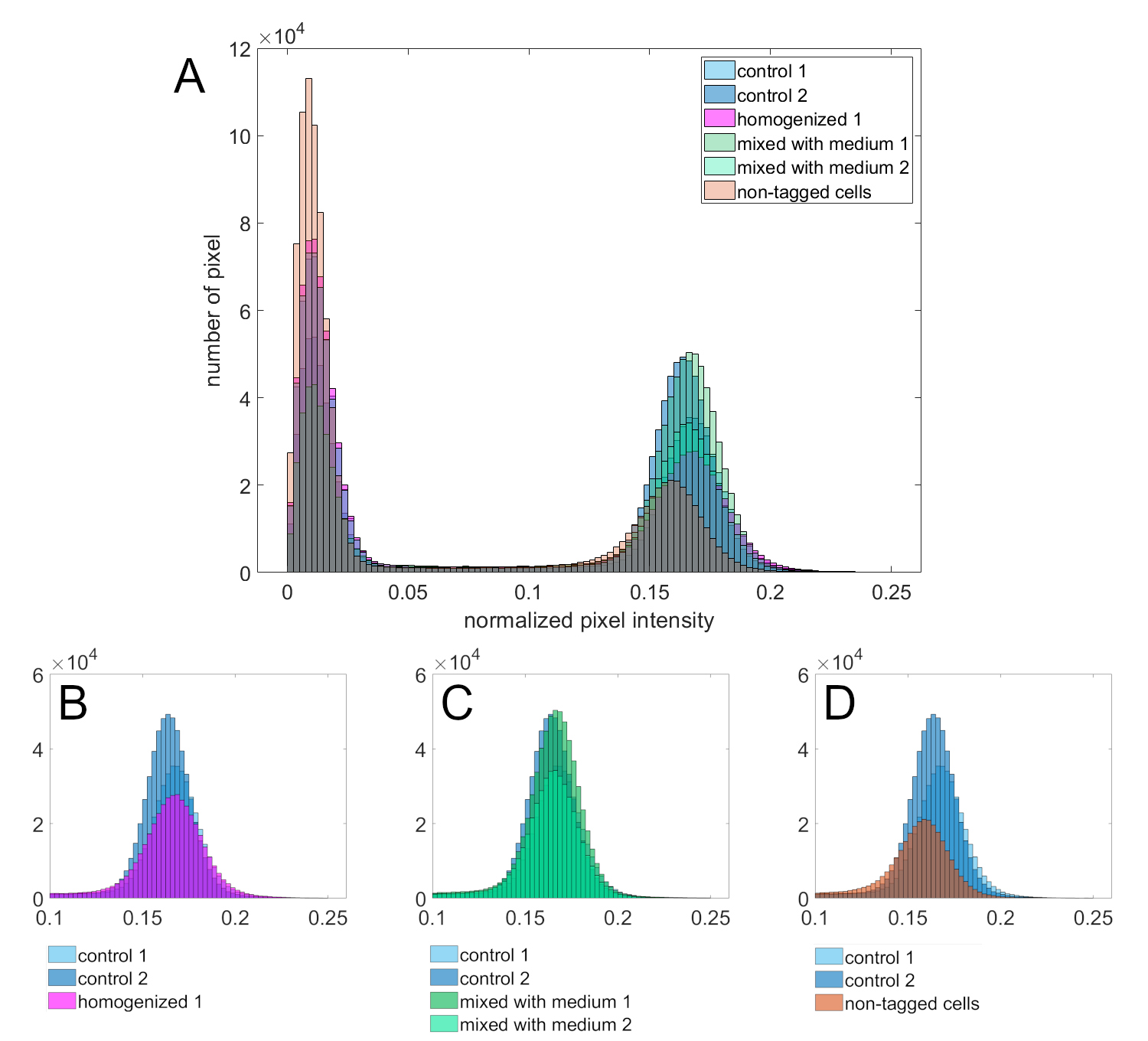

Supplement: Supplementary file 1 [file Image1.tiff]

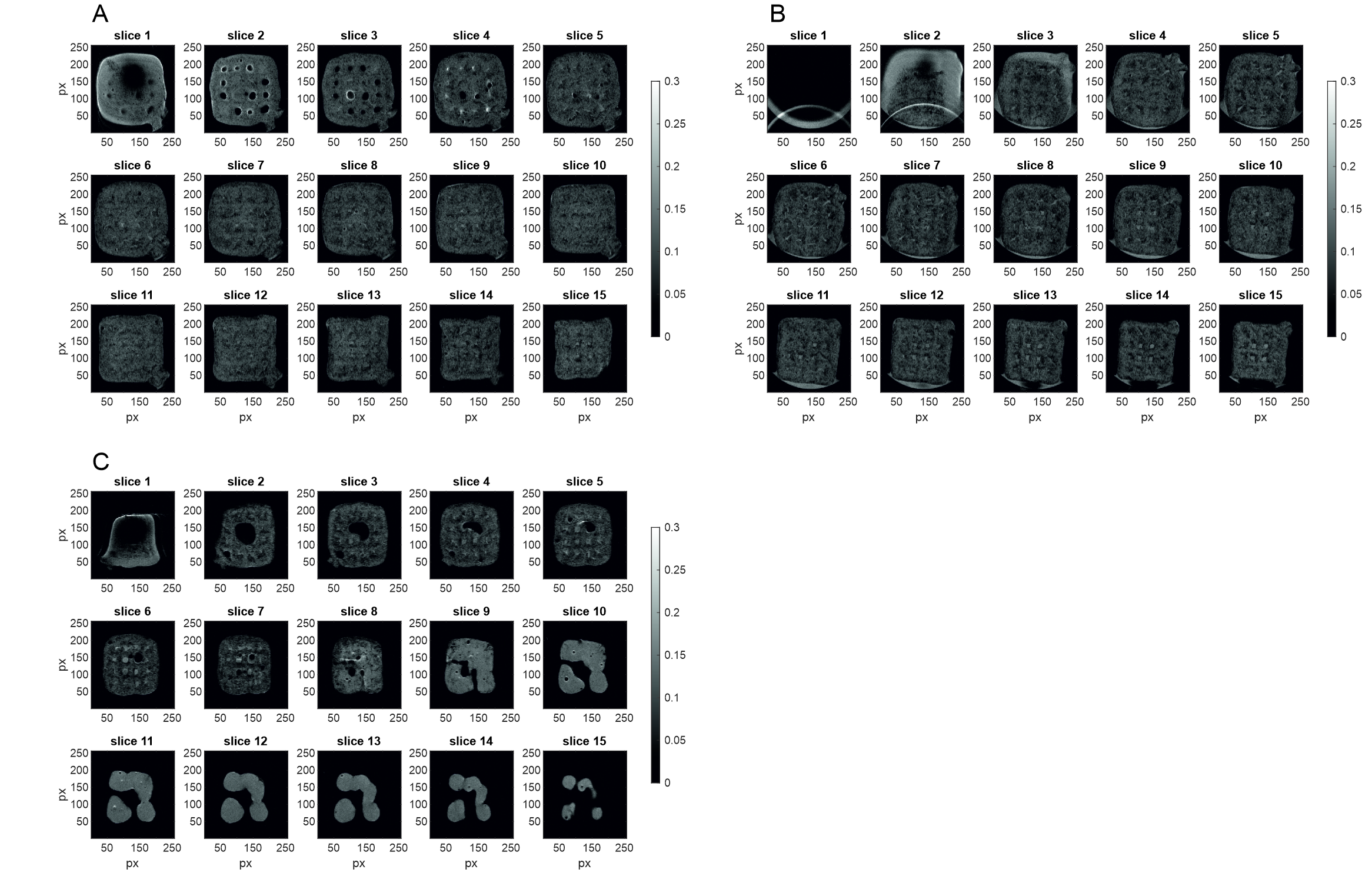

Supplement: Supplementary file 2 [file Image3.tif]

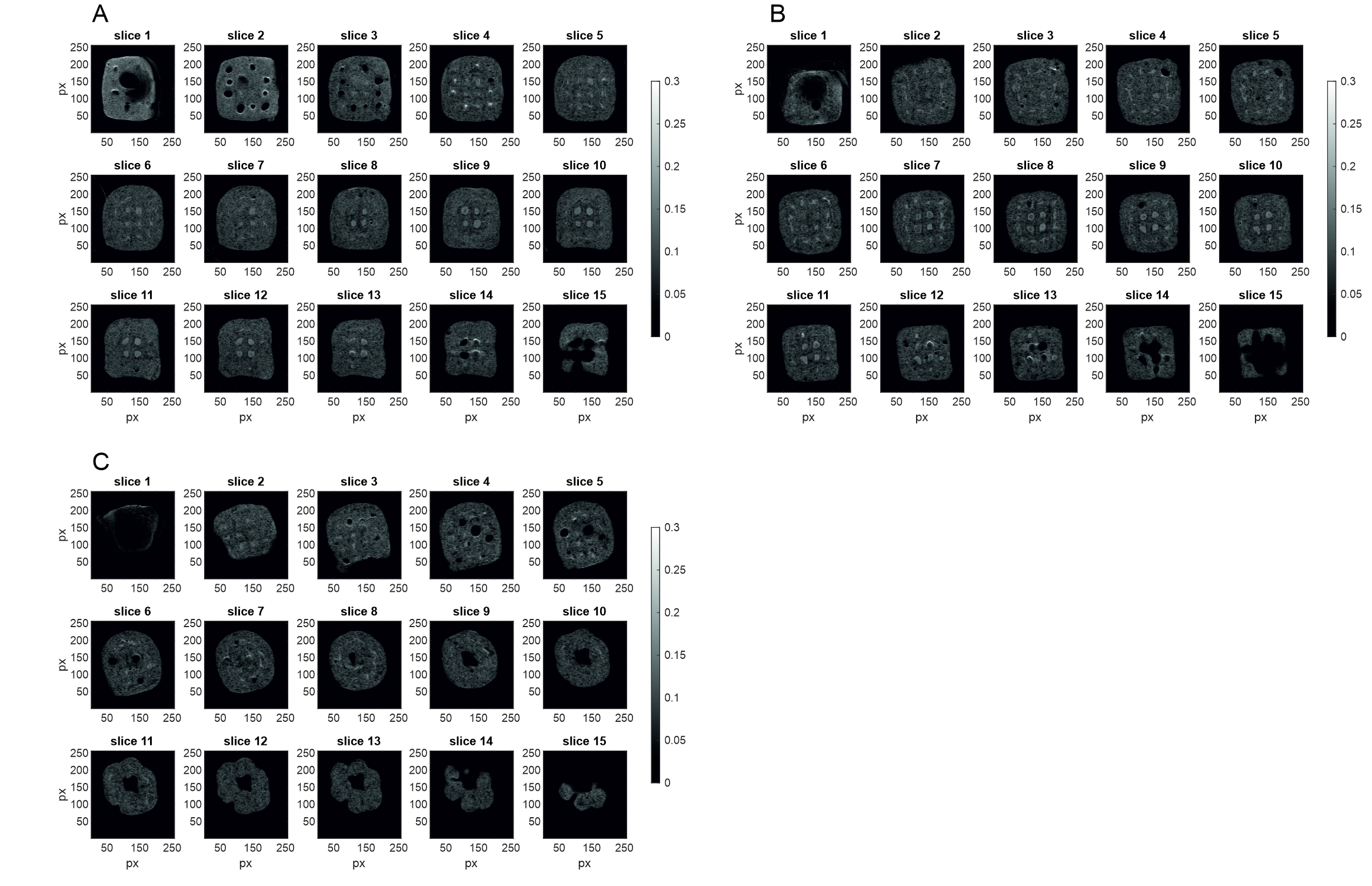

Supplement: Supplementary file 4 [file Image4.tif]

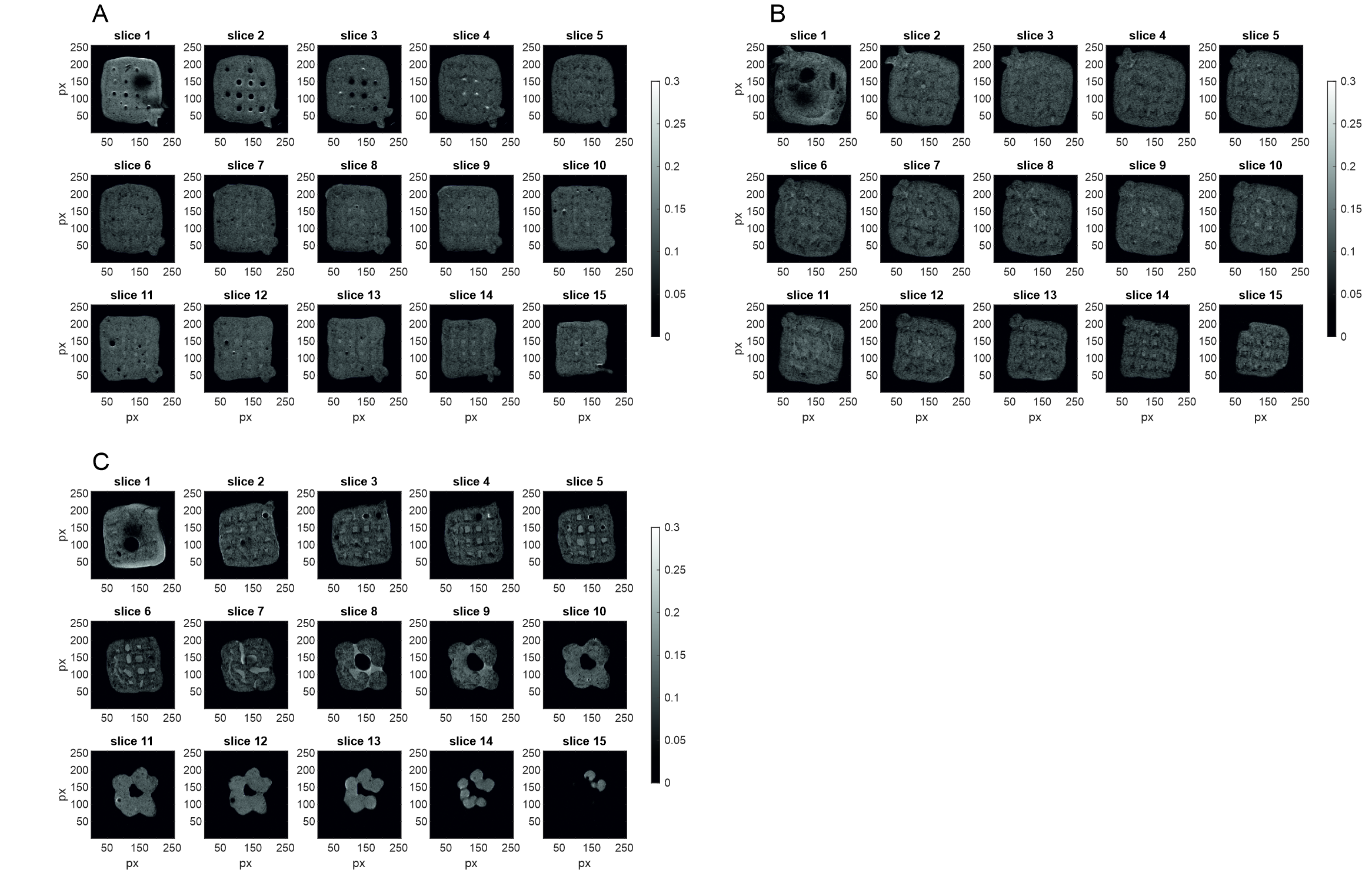

Supplement: Supplementary file 5 [file Image2.tif]
