# Supplementary material for: Virtual Reality as Tool for Bioprinting Quality Inspection: A Proof of Principle
Source: Front Bioeng Biotechnol. 2022 Jun 9;10:895842. doi: 10.3389/fbioe.2022.895842 (PMC9218671; doi:10.3389/fbioe.2022.895842)
Supplement: Supplementary file 6 [file DataSheet1.pdf]

Supplementary Data

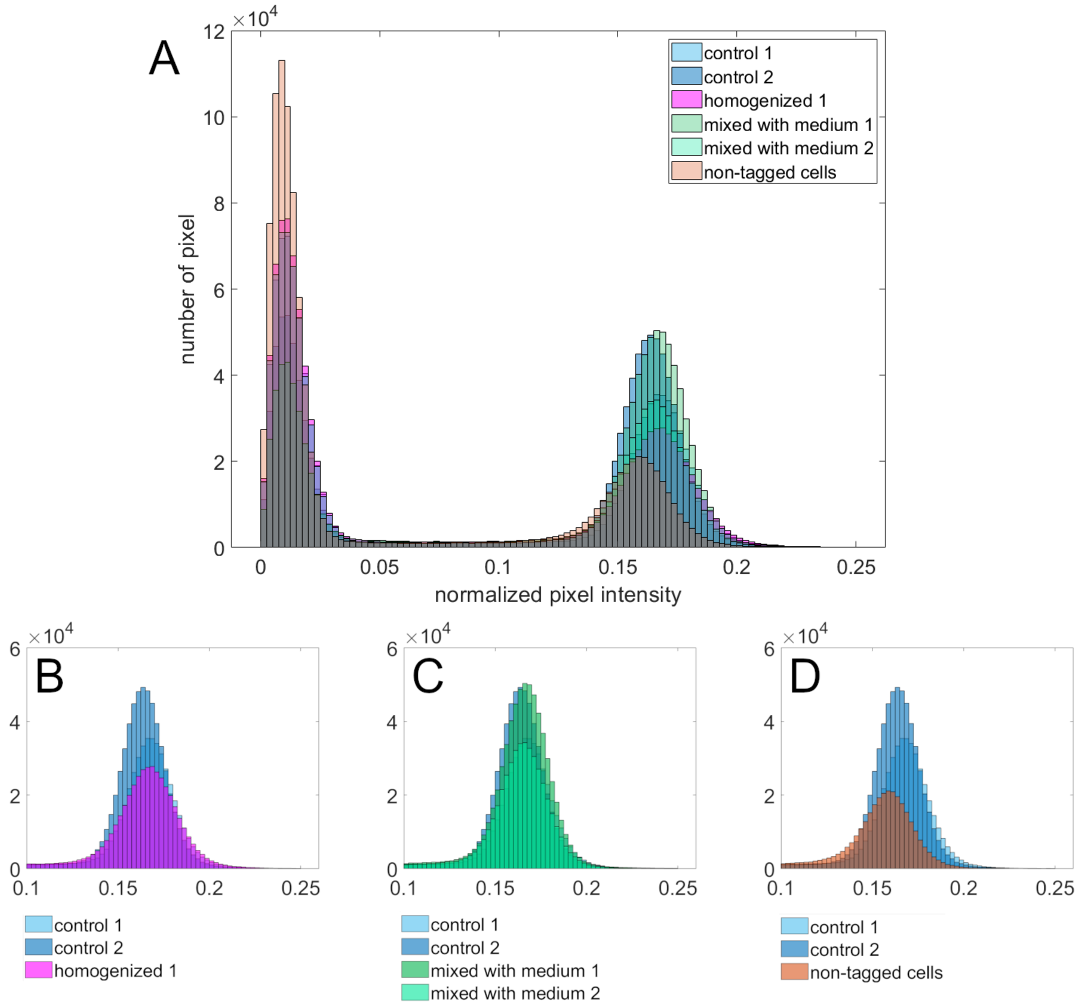

Supplementary Data 1: Histogram of reference samples with different material compositions compared to the control samples 'control 1' and 'control 2' that consist of Cellink Bioink used as received. **A)** Overlay of all reference samples. The individual compositions are explained in subfigures B-D. **B)** Cellink Bioink was homogenized in a dual asymmetric centrifuge (Speedmixer®, Hauschild GmbH & Co. KG, Hamm, Germany) for a duration of 5 min at 3500 rpm before 3D printing. **C)** An amount of 6 ml of Cellink Bioink was mixed with 600 µl of cell culture medium (Speedmixer®; 5 min; 3500 rpm) before printing with a 3 ml cartridge. MRI measurement was conducted for 2 samples printed with one cartridge of 3 ml. **D)** Bioink consisting of 3 ml of Cellink Bioink and 0.3 ml of cell suspension was prepared as described in section 2.2 of the manuscript with the exception that the NIH-3T3 fibroblasts were not tagged with NanoShuttle-PL contrast agent.

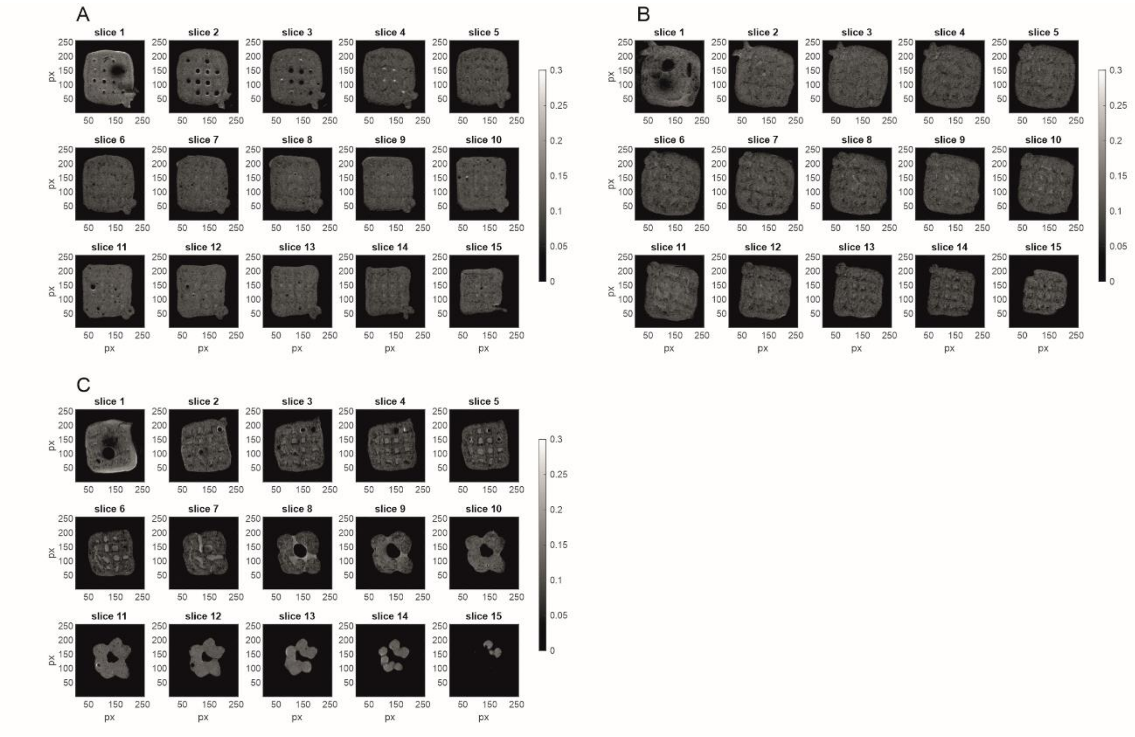

Supplementary Data 2: MRI data of the process samples run 2. Shown are the grey scale images of the 15 axial slices (z-stack) with slice 1 as bottom and slice 15 as top slice (slice thickness 0.4 mm, in plane matrix 256 x 256 px). **A)** sample 1, field of view 16 x 16 mm² **B)** sample 2, field of view 14 x 14 mm² **C)** sample 3, field of view 14 x 14 mm². The corresponding histograms of the normalized pixel intensity are shown in Figure 3 B).

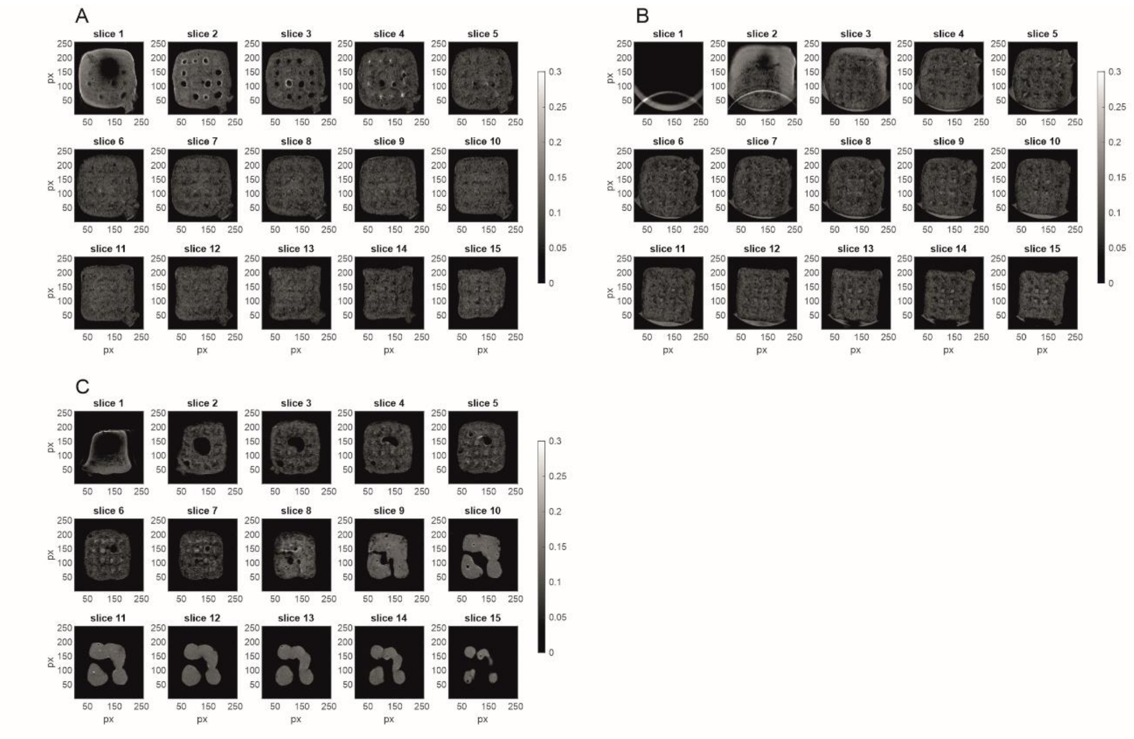

Supplementary Data 3: MRI data of the process samples run 3. Shown are the grey scale images of the 15 axial slices (z-stack) with slice 1 as bottom and slice 15 as top slice (slice thickness 0.4 mm, in plane matrix 256 x 256 px, field of view: 14 x 14 mm²). **A)** sample 1 **B)** sample 2 **C)** sample 3. The histogram of the normalized pixel intensity of samples 1 – 3 is shown in Figure 3 C).

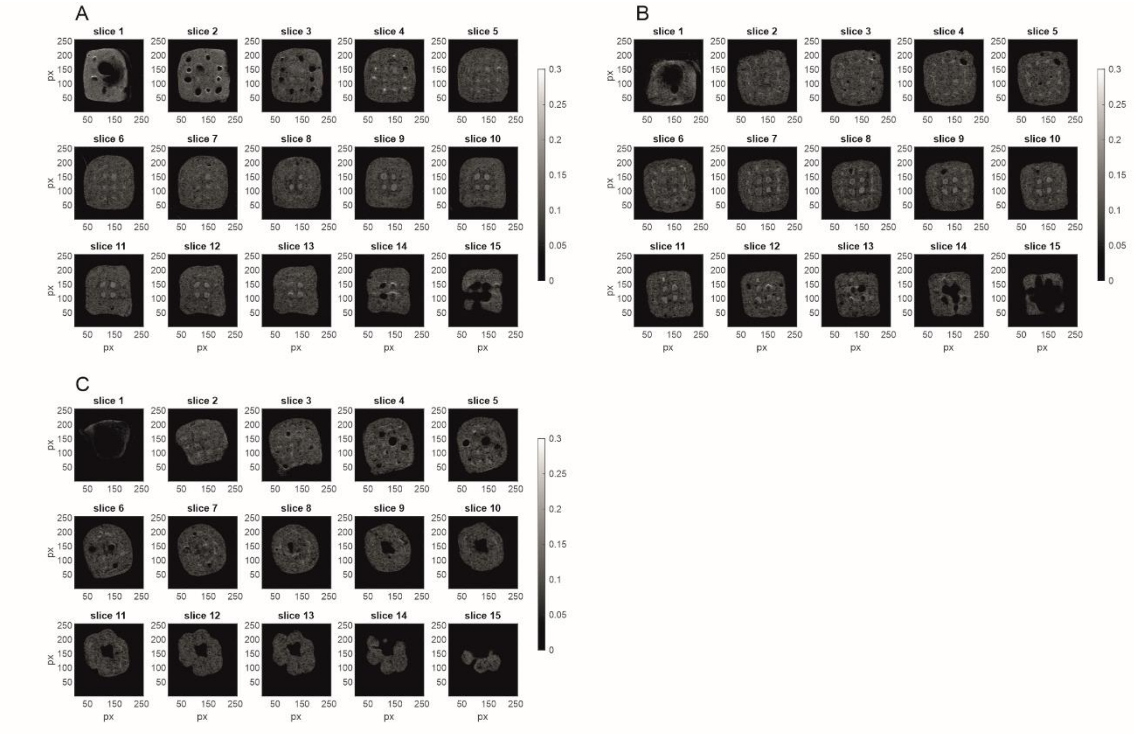

Supplementary Data 4: MRI data of the process samples run 4. Shown are the grey scale images of the 15 axial slices (z-stack) with slice 1 as bottom and slice 15 as top slice (slice thickness 0.4 mm, in plane matrix 256 x 256 px, field of view: 14 x 14 mm²). **A)** sample 1 **B)** sample 2 **C)** sample 3. The histogram of the normalized pixel intensity is shown in Figure 3 D).

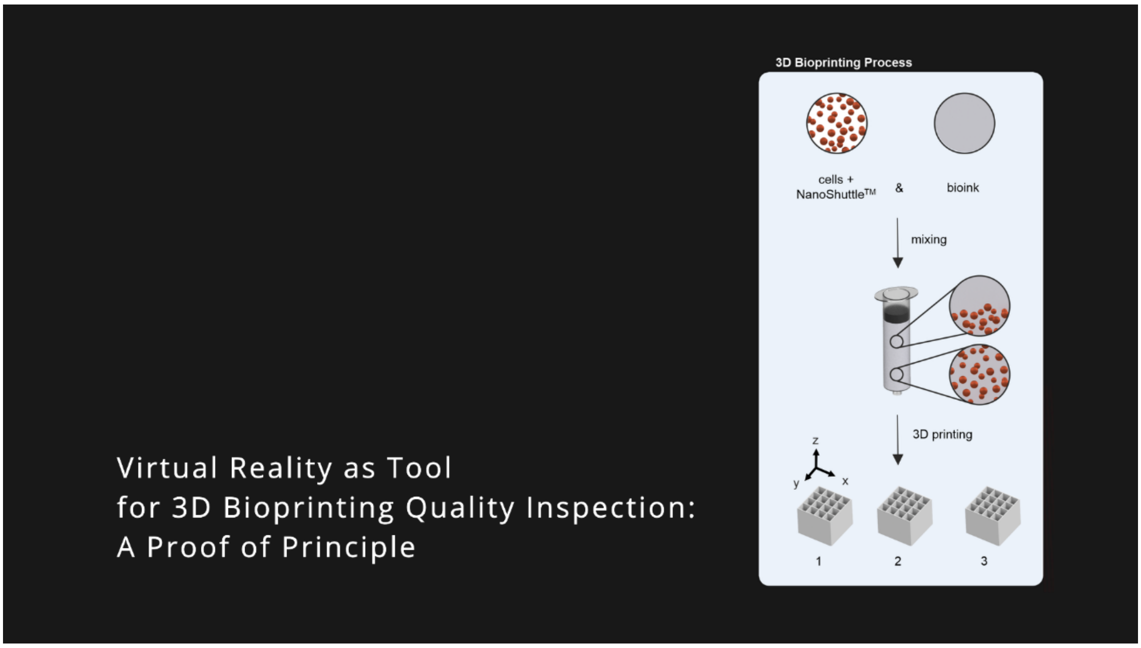

Supplementary Data 5: Video of a recorded VR session. Shown is the 3D data from control 1 and the process samples 1 – 3 (run 1). The video is edited for storytelling purpose: indication of sample and channel in the bottom left corner; indication of the cQA cell distribution in the top right corner. Each sample is visualized separately in rotation. Channels (RGB) are shown overlaid and subsequently switched off (RGB = red, green, blue; RG = red, green; R = red).

Supplementary Table 1: Example allocating the used commands for the 2D Image Analysis (section 2.4) in a sequential way. The individual form of the code options is depending on the MATLAB version and installed toolboxes.

No.	Step	Software	Comment
1)	load MRI data (.mat) into MATLAB	MATLAB 2020a (The MathWorks, Inc., Natick, 196 USA)	open the sample(s) raw data matrix of grayscale values using ‘load’
2)	metadata	MATLAB 2020a (The MathWorks, Inc., Natick, 196 USA)	define the experimental dimension of field of view as matrix, for example 'field_of_view = [12]; ', where the value 12 represents a field of view of 12 x 12 mm
3)	preview MRI slices	MATLAB 2020a (The MathWorks, Inc., Natick, 196 USA)	use the commands ‘figure’ and ‘subplot’ with the colormap ‘gray’
4)	normalize pixel intensity with regard to field of view	MATLAB 2020a (The MathWorks, Inc., Natick, 196 USA)	scale individual sample matrix with arithmetic operator, for example ‘data_scaled(:,:,:) = data(:,:,:)/(field_of_view(j))^2;’
5)	histogram	MATLAB 2020a (The MathWorks, Inc., Natick, 196 USA)	create a histogram using ‘histogram’ and adapt the depicted graph with ‘BinLimits’, and ‘BinWidth’

Supplementary Table 2: Example allocating the used commands for the 3D Image Analysis (section 2.5) and used software in a sequential way. The individual form of the code options is depending on the MATLAB version and installed toolboxes. A detailed explanation of the transfer from RGB z-stack to NIfTI for compatibility with ConfocalVR can be found in Stefani et al., 2018.

No.	Step	Software	Comment
1)	data normalization (analogue to 2D Image analysis)	MATLAB 2020a (The MathWorks, Inc., Natick, 196 USA)	scale individual sample matrix with arithmetic operator, for example 'data_scaled(:,:,:) = data(:,:,:)/(field_of_view(j))^2;'
2)	generate pseudo color images (RGB)	MATLAB 2020a (The MathWorks, Inc., Natick, 196 USA)	using the command 'imagesc' and safe each image of the stack as tif image<br>use the same 'caxis' and RGB colormap for each image, check that the image is not rotated, resized or otherwise compromised, no ticks or labels should be present
3)	re-stack RGB images	MATLAB 2020a (The MathWorks, Inc., Natick, 196 USA)	re-stack the pseudo color tif images into a new matrix using the command 'imwrite'
4)	convert RGB z-stack to NIfTI	ImageJ (National Institutes of Health [NIH], Bethesda, MD; available on (https://imagej.nih.gov/ij/)	as described in Stefani et al., 2018 (fig. 1):<br>load image stack with Bio-format plugin<br>choose visual representation (color channel 'red, green, blue')<br>'convert to' RGB color image stack<br>Save as NIfTI data transfer format to open in ConfocalVR
5)	ConfocalVR	ConfocalVR 3.2 218 (Immersive Science LLC, Newcastle, WA USA)	As described in Stefani et al., 2018:<br>rotation of virtual sample and RGB channel (de)activation in virtual control panel
